# Synergy of therapeutic heterologous prime-boost hepatitis B vaccination with CpG-application to improve immune control of persistent HBV infection

**DOI:** 10.1038/s41598-019-47149-w

**Published:** 2019-07-25

**Authors:** Anna D. Kosinska, Abdul Moeed, Nina Kallin, Julia Festag, Jinpeng Su, Katja Steiger, Marie-Louise Michel, Ulrike Protzer, Percy A. Knolle

**Affiliations:** 1 Institute of Virology, Technical University of Munich, Munich, Germany; 2 Helmholtz Centre Munich, Munich, Germany; 3 Institute of Molecular Immunology and Experimental Oncology, Technical University of Munich, Munich, Germany; 4 Institute of Pathology, Technical University of Munich, Munich, Germany; 5 Institute Pasteur, Paris, France; 6 German Center for Infection Research (DZIF), Partner site München, Munich, Germany

**Keywords:** Protein vaccines, Immunization, Hepatitis B

## Abstract

Therapeutic vaccination against chronic hepatitis B must overcome high viral antigen load and local regulatory mechanisms that promote immune-tolerance in the liver and curtail hepatitis B virus (HBV)-specific CD8 T cell immunity. Here, we report that therapeutic heterologous HBcore-protein-prime/Modified-Vaccinia-Virus-Ankara (MVA-HBcore) boost vaccination followed by CpG-application augmented vaccine-induced HBcAg-specific CD8 T cell-function in the liver. In HBV-transgenic as well as AAV-HBV-transduced mice with persistent high-level HBV-replication, the combination of therapeutic vaccination with subsequent CpG-application was synergistic to generate more potent HBV-specific CD8 T cell immunity that improved control of hepatocytes replicating HBV.

## Introduction

Chronic hepatitis B affects more than 250 million persons worldwide^1^, and results from insufficient immune control of hepatitis B virus (HBV) infection^2,3^, for which no curative direct anti-viral pharmacological treatment is available^4^. Immune control of chronic hepatitis B, however, can spontaneously occur at a low frequency of 0.5% of patients per year^5,6^. This indicates that inducing HBV-specific immunity by vaccination may be a promising immune-therapeutic approach^7^. Therapeutic vaccination against persistent hepatitis B infection has been intensively studied over the last years, but so far with no breakthrough success^8^. Heterologous prime-boost vaccination has been suspected to be superior to other vaccination approaches to elicit strong antigen-specific immunity and overcome existing immune tolerance, making it an interesting option for therapeutic vaccination^9^. We recently developed a therapeutic heterologous prime-boost vaccination protocol based on a prime with particulate HBV antigen followed by boost-vaccination with a recombinant modified vaccinia virus Ankara (MVA) expressing HBV-antigens^10^, which compared to other currently employed therapeutic vaccination strategies induces strong antiviral immunity^7^. This therapeutic prime-boost vaccine elicits both, anti-HBs seroconversion and strong HBcAg-specific CD8 T cell immunity^10^. It broke HBV-specific immune tolerance in HBV-transgenic mice with low to medium levels of HBV antigen, but in the presence of high HBV antigen levels, the therapeutic vaccine failed to induce HBcAg-specific CD8 T cell immunity^10^. Since CD8 T cell immunity to HBV is critical for control of HBV infection^11^, we reasoned that enhancing HBcAg-specific CD8 T cell immunity in the liver should increase the efficiency of therapeutic heterologous prime-boost vaccination against chronic HBV infection.

The unique tolerogenic hepatic microenvironment^12^ is involved in local skewing of virus-specific CD8 T cell responses and supports the development of persistent infection^13^. However, local inflammation in the liver triggered by the TLR9-ligand CpG, enhances the development of virus-specific CD8 T cell immunity. Inflammatory monocytes, that accumulate in the liver upon CpG injection, introduce an immunogenic microenvironment through formation of cocoon-like structures termed iMATEs (intrahepatic myeloid cell aggregates associated with T cell expansion) and provide co-stimulatory signals through OX40 and CD28 to trigger local hepatic CD8 T cell-activation and proliferation^14^. We reasoned that heterologous prime-boost therapeutic vaccination leading to priming of HBcAg-specific CD8 T cells within lymphoid tissues would be complemented by CpG-injection to enhance vaccine-induced CD8 T cell immunity locally in the liver.

## Results

### In high-titer HBV transgenic mice, CpG-injection improves immune control after heterologous prime-boost vaccination

**Figure 1:**
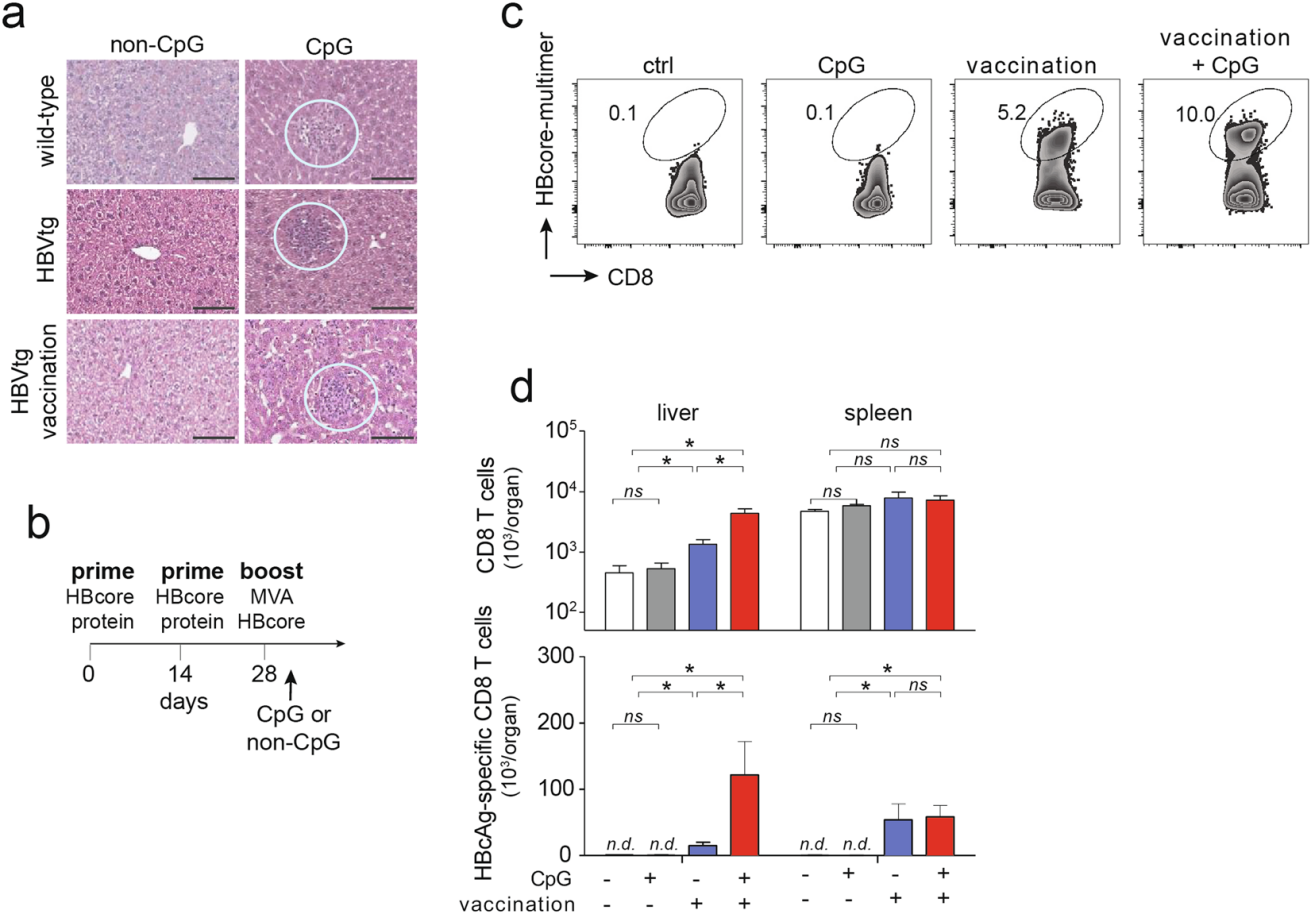
Intravenous CpG-injection leads to iMATE-formation in HBV-transgenic mice and expansion of HBcAg-specific CD8 T cells in the liver. (**a**) H&E staining of liver tissue slices detecting iMATEs at d3 after CpG-injection (white circles). (**b**) Time scheme for therapeutic heterologous prime boost vaccination against HBcAg and CpG-injection. (**c**) HBcAg-specific CD8 T cells from liver detected by HBc-specific multimer-staining using flow cytometry at d3 after CpG-injection, numbers denote percentage of total liver CD8 T cells. (**d**) Absolute numbers of total and HBcAg-specific CD8 T cells in liver and spleen at d3 after CpG-injection. Bars in (**d**) indicate mean value of *n* ≥ 3 mice per group + SEM. Statistical analyses were performed using Kruskal-Wallis test with Dunn’s multiple comparison correction. Asterisks mark statistically significant differences: *p < 0.05; *ns* – not significant; *n.d*. – not detectable

We used HBV-transgenic mice as a well-characterized preclinical model of HBV persistence^15^ to evaluate whether the combination of therapeutic heterologous prime-boost vaccination directed against HBcAg synergizes with CpG-treatment to control HBV infection. We used HBV-transgenic mice with intermediate to high HBsAg levels (50 to 450 IU/ml), where prime-boost vaccination fails to elicit strong HBV-specific CD8 T cell immunity^10^. CpG-application always led to formation of iMATEs, in wildtype C57Bl/6 mice, in HBV-transgenic mice or in vaccinated HBV-transgenic mice with high-level HBV replication (Fig. 1a). First, we determined the optimal time point for CpG application after heterologous prime-boost vaccination. CpG-application within three days after heterologous prime-boost vaccination, but not later, resulted in increased numbers of vaccination-induced HBcAg-specific CD8 T cells (Suppl. Fig. 1, for gating strategy see Suppl. Fig. 2), which led us to apply CpG within the first three days after vaccination in all further experiments (see scheme in Fig. 1b). Since HBcAg-specific CD8 T cells are key for control of HBV infection^10,16^, we determined numbers of vaccination-induced HBcAg-specific CD8 T cells in the presence or absence of CpG-application. We detected an increase in the numbers of HBcAg-specific CD8 T cells after heterologous prime-boost vaccination in HBV-transgenic mice and a further increase upon CpG-application (Fig. 1c). Of note, we also found HBcAg-specific CD8 T cells in the spleen, but these cells were not further increased in numbers after CpG-application (Fig. 1d). This finding suggested hepatic expansion of already activated HBcAg-specific CD8 T cells generated by vaccination, but not local priming of naïve CD8 T cells after CpG application^14^. We found expression of Ki67, a marker for cell proliferation, in hepatic mononuclear cells within iMATEs after CpG-injection by immunohistochemistry in vaccinated mice (Suppl. Fig. 3a). Furthermore, we detected Ki-67 expression in the majority of hepatic CD8 T cells at d3 after CpG injection by flow cytometry (Suppl. Fig. 3b). Consistent with previous reports^14,17^, application of CpG resulted in increased expression of costimulatory molecules like CD80 and CD86 as well as increased MHC-II expression in infiltrating monocytes (Suppl. Fig. 4a).

**Figure 2:**
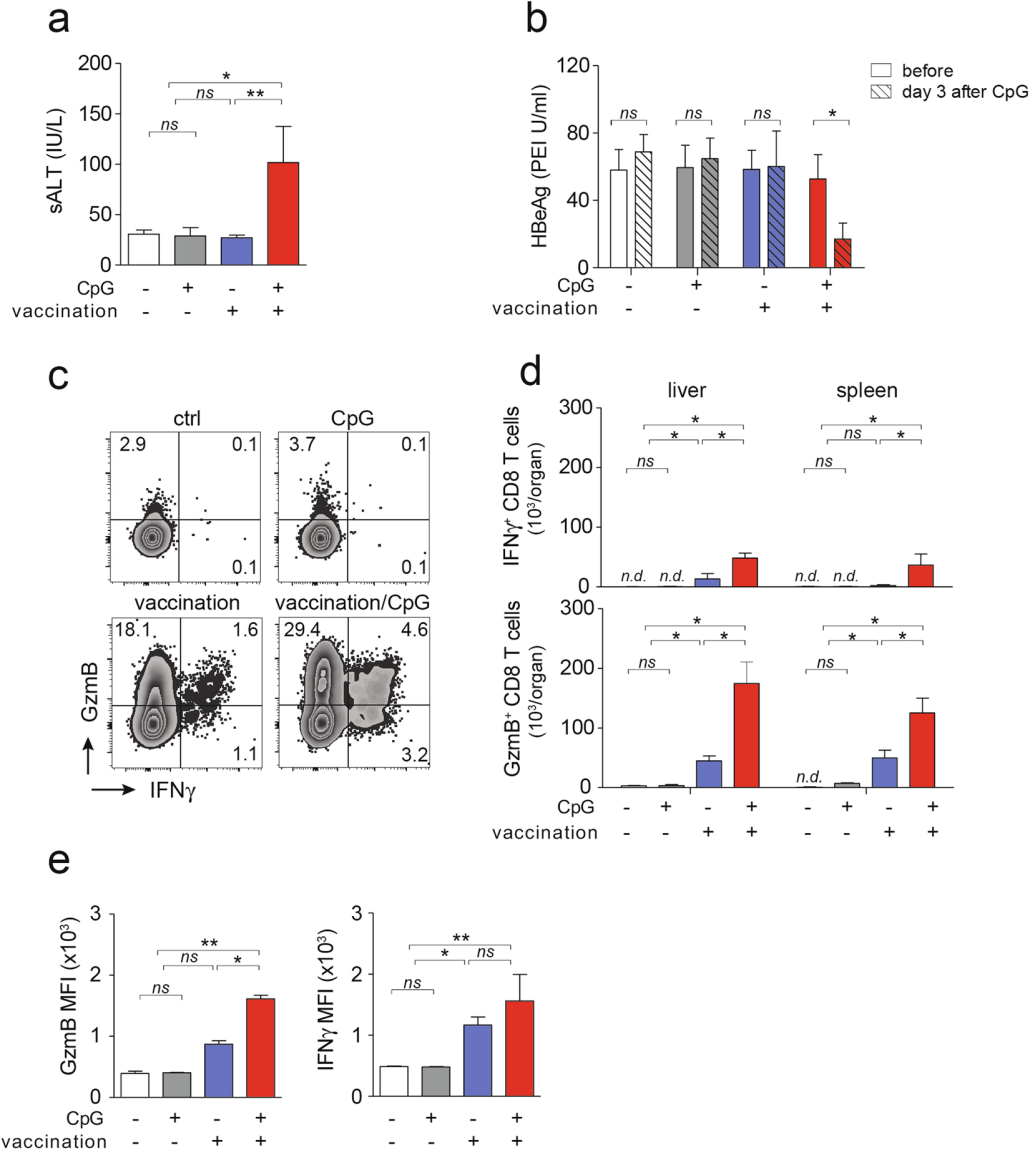
Antiviral effector function and phenotype of HBcAg-specific CD8 T cells. (**a,b**) Liver damage as determined by measurement of serum ALT at d31 after start of vaccination and d3 after CpG-injection (**a**) and reduction in serum HBeAg levels (**b**) in HBV transgenic mice. (**c**) Intracellular levels of GzmB and IFNγ in hepatic CD8 T cells following *ex vivo* restimulation with an HBcore peptide. (**d,e**) Numbers and expression levels of GzmB^+^ and IFNγ^+^ in HBcAg-specific CD8 T cells after *ex vivo* restimulation. Bars in (**a**,**b**,**d**,**e**) indicate mean value of *n* ≥ 3 mice + SEM. Statistical analyses were performed using (**a**,**d**) Kruskal-Wallis test with Dunn’s multiple comparison correction, (**b**) paired *t*-test and (**e**) Mann-Whitney test. Asterisks mark statistically significant differences: *p < 0.05; *ns* – not significant; *n.d*. – not detectable

Following heterologous prime-boost vaccination and subsequent CpG-injection in HBV-transgenic mice, we observed development of increased serum ALT levels compared to mice receiving vaccination alone, indicating increased HBcAg-specific CD8 T cell immunity in the liver (Fig. 2a). Consistent with previous results^10^, in the presence of high HBV expression levels in HBV-transgenic mice, heterologous prime-boost vaccination alone did not reduce serum HBeAg levels, neither did CpG application alone (Fig. 2b). After sequential application of heterologous prime-boost vaccination and CpG injection, however, we found a significant decline in serum HBeAg-levels (Fig. 2b). These findings suggested that HBcAg-specific CD8 T cells locally expanded in the liver within iMATEs after CpG-injection, and controlled HBV replication in infected hepatocytes. We next determined the functionality of these HBcAg-specific CD8 T cells from liver or spleen following *ex vivo* restimulation. HBcAg-specific CD8 T cells isolated from liver or spleen of HBV-transgenic mice after heterologous prime-boost vaccination expressed IFNγ following re-stimulation with an HBcore peptide (Fig. 2c). After sequential application of vaccination and CpG, numbers of IFNγ-producing HBcAg-specific CD8 T cells were 4-fold increased, as were numbers of GzmB-expressing CD8 T cells, now constituting almost 30 percent of all hepatic CD8 T cells (Fig. 2c,d). Of note, vaccine-induced CD8 T cells expressed higher levels of GzmB but not IFNγ per cell (Fig. 2e) suggesting enhanced cytotoxic effector function.

**Figure 3:**
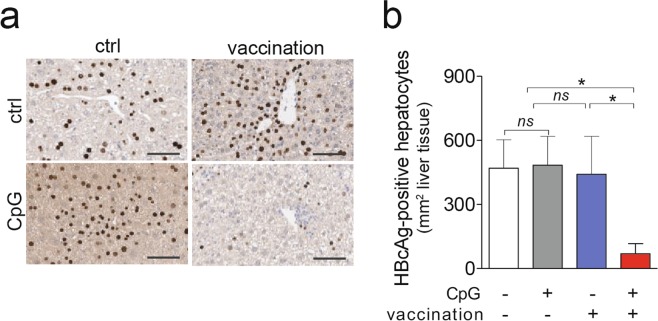
Effect of heterologous prime-boost vaccination in combination with CpG injection on control of hepatocytes expressing HBcAg in HBV-transgenic mice. (**a**) Liver immunohistochemistry detecting HBcAg-positive hepatocytes. (**b**) Quantification from (**a**). Bars in (**b**) indicate mean value of *n* ≥ 3 mice per group + SEM. Statistical analyses was performed using Kruskal-Wallis test with Dunn’s multiple comparison correction. Asterisks mark statistically significant differences: *p < 0.05; *ns* – not significant.

Consistent with enhanced effector function and release of anti-viral cytokines of HBcAg-specific CD8 T cells, liver immune-histochemistry revealed that sequential application of vaccination together with CpG but not vaccination alone led to a 6-fold reduction in numbers of HBcAg-positive hepatocytes (Fig. 3a,b). The relatively low sALT values (see Fig. 2a) support the notion that reduction of HBcore-positive hepatocytes may also have been achieved by non-cytolytic activity of cytokines. Together, these results indicated that CpG-application acted in a synergistic fashion with therapeutic vaccination to augment both, numbers and functionality of vaccine-induced HBcAg-specific CD8 T cells in HBV-transgenic mice.

### Control of infection in high-titer AAV-HBV infected mice after heterologous prime-boost vaccination followed by CpG injection

**Figure 4:**
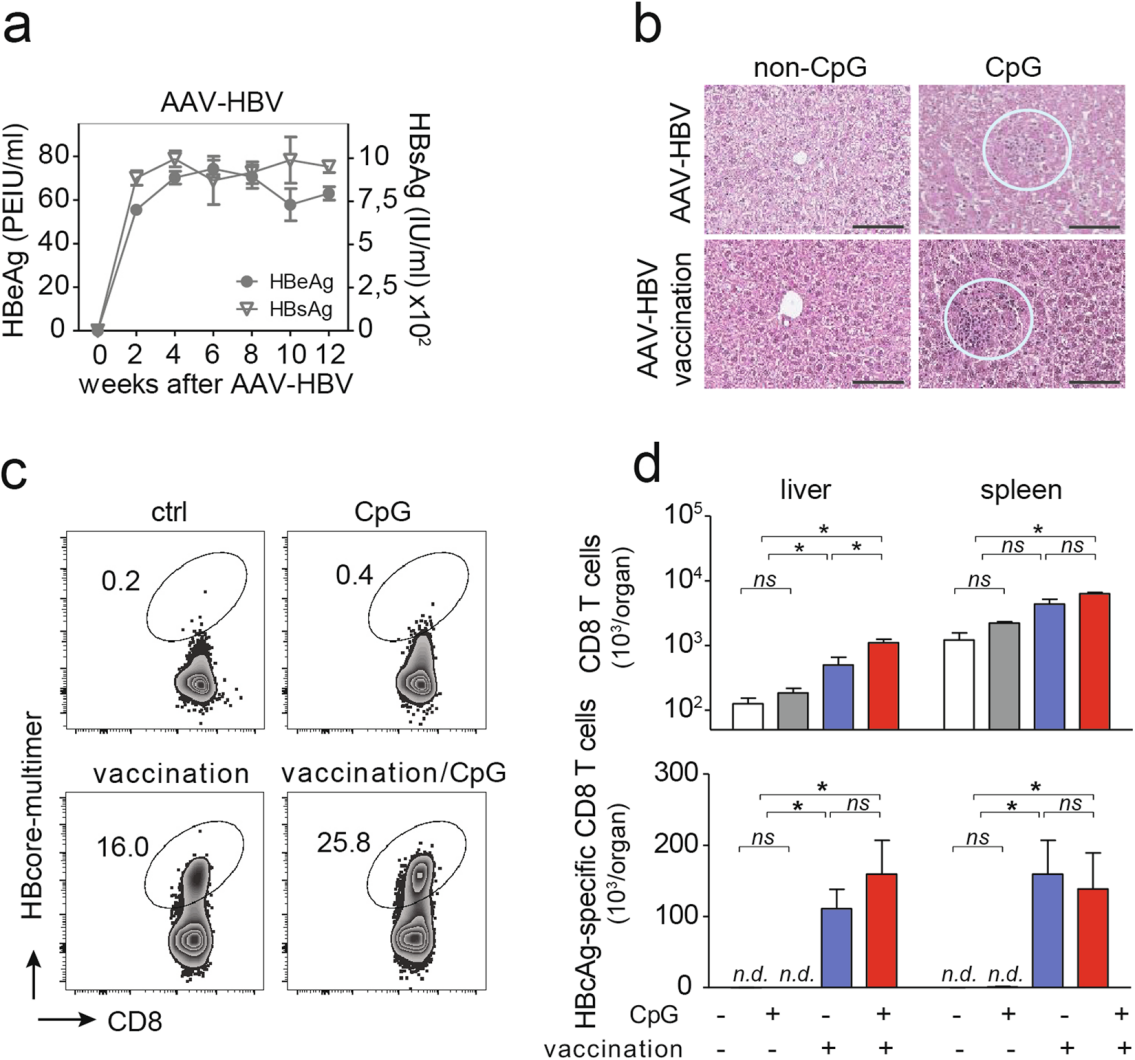
Vaccination-induced generation of HBcAg-specific CD8 T cells in the AAV-HBV model. (**a**) Time kinetics of serum HBV antigen levels after AAV-HBV transduction of C57Bl/6 mice. (**b**) H&E staining of liver slices detecting iMATEs following CpG-injection (white circles). (**c**) Hepatic HBcAg-specific CD8 T cells detected by HBcAg-specific multimer-staining and detection by flow cytometry, numbers denote percentage of total hepatic CD8 T cells. (**d**) Absolute numbers of total and HBcAg-specific CD8 T cells in liver and spleen. Bars in (d) indicate mean value of *n* ≥ 3 mice per group + SEM. Statistical analyses were performed using Kruskal-Wallis test with Dunn’s multiple comparison correction. Asterisks mark statistically significant differences: *p < 0.05; *ns* – not significant; *n.d*. – not detectable.

Since elimination of an HBV-transgene from hepatocytes is not possible, we employed AAV-HBV infection to deliver HBV genomes into murine hepatocytes *in vivo*^18^, which leads to persistent HBV replication in hepatocytes^18^. This preclinical model system of persistent HBV replication can be used to characterize whether vaccine-induced HBV-specific CD8 T cells successfully eliminated HBV-replicating hepatocytes *in vivo*. After AAV-HBV-transduction, high serum HBeAg and HBsAg levels were detected, that remained stable until the end of the observation period twelve weeks later indicating persistent HBV replication (Fig. 4a). These HBsAg levels (200–1200 IU/ml) were comparable to the intermediate to high antigen level groups previously reported^10^ where heterologous prime-boost vaccination fails to elicit strong HBV-specific CD8 T cell immunity. Of note, no anti-HBs or anti-HBe antibodies were detected in these mice before start of vaccination (Suppl. Fig. 5a). In AAV-HBV-transduced mice, CpG-application triggered iMATE-formation independent of heterologous prime-boost vaccination (Fig. 4b). As in HBV-transgenic mice, therapeutic vaccination induced HBcAg-specific CD8 T cells in AAV-HBV transduced mice (Fig. 4c,d). Consistent with an hepatic expansion of vaccine-induced T cells, we found more HBcAg-specific CD8 T cells in the livers but not spleens in AAV-HBV transduced mice after sequential application of heterologous prime-boost vaccination and CpG injection, although this did not reach statistical significance (Fig. 4c,d). Expansion of CD8 T cells after CpG injection in AAV-HBV transduced mice was confirmed by detection of Ki67 expression in hepatic CD8 T cells (Suppl. Fig. 3c,d). Compared to prime-boost vaccination in HBV-transgenic mice, that yielded only very few HBcAg-specific CD8 T cells (see Fig. 1), we detected more HBcAg-specific CD8 T cells in AAV-HBV-transduced mice after vaccination (Fig. 4c,d). This may be the result of the more stringent deletion of HBV-specific T cells through central tolerance in HBV-transgenic mice^19^, rather than changes in expression of co-stimulatory molecules on monocytes infiltrating the liver and forming iMATEs after CpG injection (Suppl. Fig. 4b). Again, CpG-application without prior vaccination, did not lead to any measurable increase in HBcAg-specific CD8 T cells (Fig. 4c,d).

**Figure 5:**
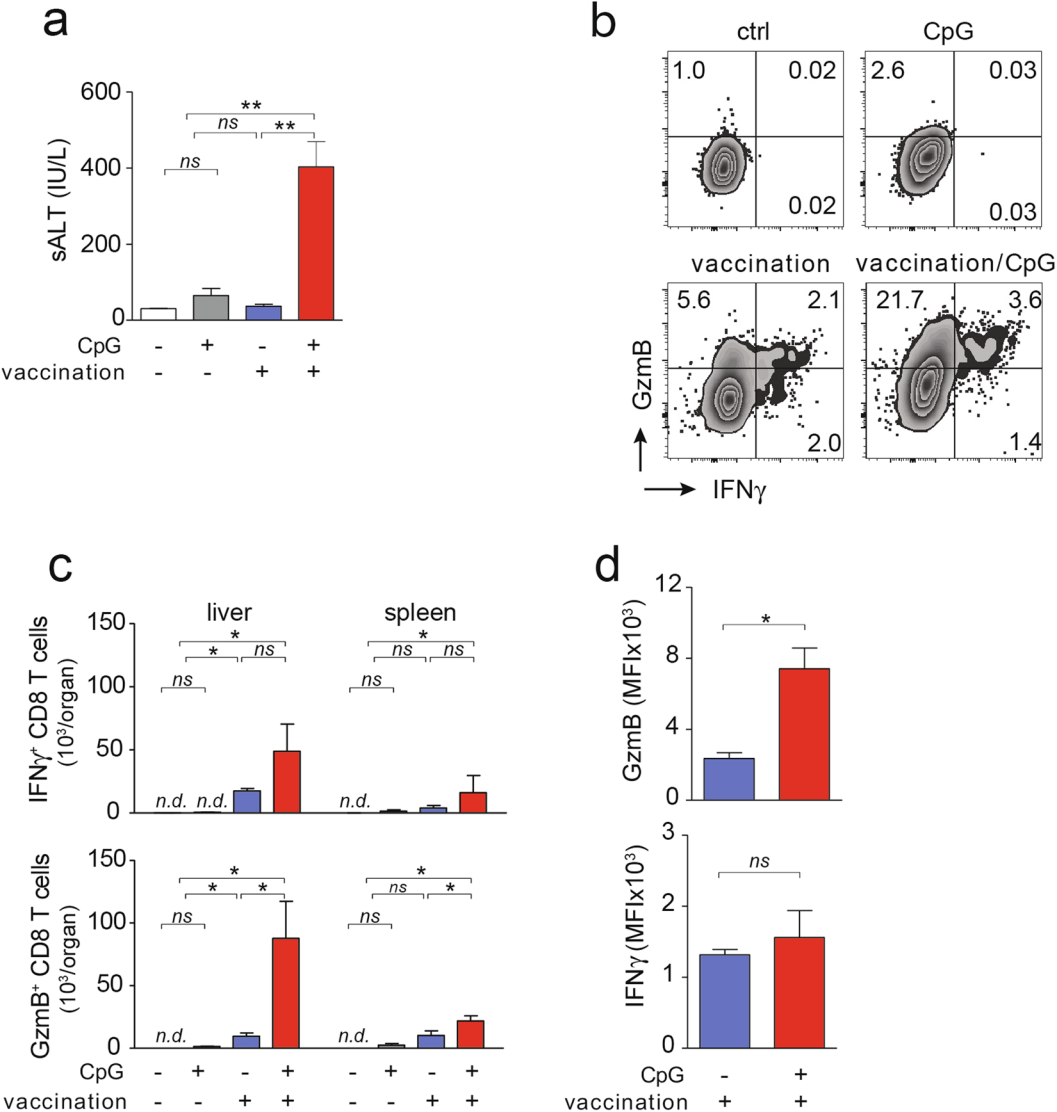
Antiviral effector function and phenotype of HBcAg-specific CD8 T cells in AAV-HBV replicating mice after therapeutic vaccination in combination with CpG injection. (**a**) Liver damage as determined serum ALT at d31 after start of vaccination and d3 after CpG-injection. (**b**) Intracellular staining for GzmB and IFNγ in hepatic CD8 T cells following *ex vivo* restimulation with an HBcore peptide. (**c,d**) Numbers and expression levels of GzmB and IFNγ in HBcAg-specific CD8 T cells after *ex vivo* restimulation. Bars in (a,c,d) indicate mean value of *n* ≥ 3 mice per group + SEM. Statistical analyses was performed using (**a**,**d**) Kruskal-Wallis test with Dunn’s multiple comparison correction and (**d**) unpaired *t*-test. Asterisks mark statistically significant differences: *p < 0.05; *ns* – not significant; *n.d*. – not detectable.

Only the sequential application of heterologous prime-boost vaccination and CpG injection but neither CpG-injection nor vaccination alone resulted in increased sALT levels nine weeks after AAV-HBV transduction (Fig. 5a). Numbers of IFNγ-producing and GzmB-expressing HBcAg-specific CD8 T cells were higher after heterologous prime-boost vaccination in AAV-HBV infected mice, were further increased after CpG-injection, and were higher in liver than in spleen (Fig. 5b,c). Furthermore, GzmB but not IFNγ levels per HBcAg-specific CD8 T cell were significantly higher when heterologous prime-boost vaccination was followed by CpG-injection (Fig. 5d). Thus, also in AAV-HBV-transduced mice with high-level HBV replication, CpG-application after vaccination increased both, numbers and of functionality of vaccine-induced HBcAg-specific CD8 T cells.

**Figure 6:**
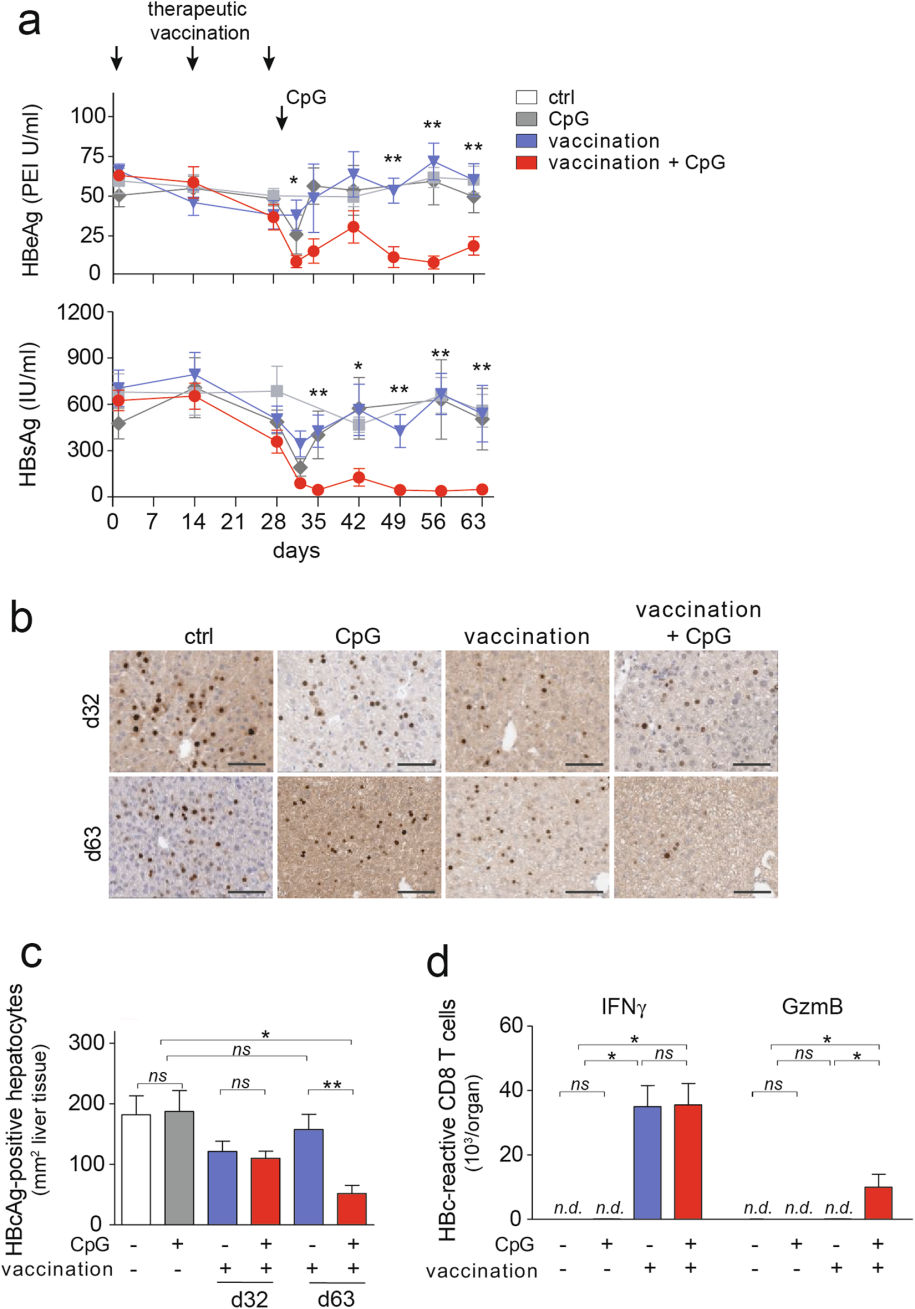
Control of numbers of HBcAg-expressing hepatocytes after therapeutic vaccination ± CpG injection in AAV-HBV-infected mice. (**a**) Time course of serum HBeAg and HBsAg levels after therapeutic heterologous prime-boost vaccination. (**b**,**c**) Liver immunohistochemistry of HBcAg-expressing hepatocytes at d32 and d63 after start of vaccination (d3 and d34 after CpG-injection) and quantification. (**d**) Flow cytometric evaluation of intracellular GzmB and IFNγ in HBcAg-specific CD8 T cells after restimulation *ex vivo*. Bars in (**a**,**c**,**d**) indicate mean value of *n* ≥ 3 mice per group + SEM. Statistical analyses was performed using (**a**) 2-way ANOVA with Tukey’s multiple comparison correction and (**c**,**d**) Kruskal-Wallis test with Dunn’s multiple comparison correction. Asterisks mark statistically significant differences: *p < 0.05; **p < 0.01; *ns* – not significant; *n.d*. – not detectable.

We then followed AAV-HBV infected mice after vaccination ± CpG-injection for five weeks. Heterologous prime-boost vaccination alone led to a transient decrease of serum HBeAg-levels that was not very much different from non-vaccinated mice (Fig. 6a). In contrast, when vaccination was followed by CpG-injection serum HBeAg levels remained low over the entire observation period (Fig. 6a). Similarly, HBsAg levels were controlled after the combination of vaccination and CpG-injection (Fig. 6a), even in the absence of seroconversion to anti-HBs or anti-HBe (Suppl. Fig. 5b,c). Liver immunohistochemistry revealed that shortly after CpG-injection in vaccinated AAV-HBV infected mice (d32 after initial start of vaccination) there was no difference in numbers of HBcAg-expressing hepatocytes compared to mice that did not receive CpG (Fig. 6b,c). However, more than four weeks later (i.e., d63 after start of vaccination) following sequential application of vaccination and CpG-injection, numbers of HBcAg-expressing hepatocytes were reduced by 80%, while no control of HBcAg-expressing hepatocytes was observed after vaccination alone (Fig. 6b,c). At this late time point, only GzmB-expressing HBcAg-reactive CD8 T-cells were still increased in AAV-HBV-infected mice that received a combinatorial treatment of therapeutic vaccination followed by CpG-injection (Fig. 6d), suggesting that detection of GzmB expression in virus-specific CD8 T cells may allow for prediction of immune control of HBV-replicating hepatocytes.

## Discussion

Here we report that therapeutic heterologous protein-prime/MVA-vector-boost vaccination against hepatitis B synergizes with CpG-application to enhance numbers and functionality of HBcAg-specific CD8 T-cells in presence of high HBV-antigen levels in two preclinical models of persistent HBV infection, i.e. the AAV-HBV model and HBV-transgenic mice. High viral antigen levels prevented formation and expansion of HBcAg-specific CD8 T cells, that are required to gain control of HBV infection^11^. Cytokines released from T cells like IFNγ/TNF control HBV-antigen expression through degradation of the HBV persistence form in infected hepatocytes^20,21^. Cytokines also exert non-cytolytic control of HBV antigen expression in preclinical models such as the HBV transgenic mouse^22^ and may thereby account in part for the rapid decline of serum HBeAg-levels after vaccination in combination with CpG-injection, which leads to release of cytokines from T cells^14^.

Long-term control of persistent HBV-infection, however, relies on reduction of the numbers of HBV-infected hepatocytes, which may either be achieved through degradation of the HBV persistence form or elimination of infected and HBV-replicating hepatocytes. Here, we demonstrate that the combination of therapeutic heterologous prime-boost vaccination with CpG-injection led to strong reduction of the numbers of HBcAg-expressing hepatocytes together with local hepatic expansion of vaccination-induced HBcAg-specific CD8 T cells. This suggests that CpG-injection, which functions to induce formation of iMATEs and to locally expand already activated CD8 T cells in the liver by providing costimulatory signals^14^, can be employed to increase the efficacy of therapeutic vaccination against HBV even in the presence of high-level viral antigens, which otherwise prevent induction of protective immunity. However, it is also possible that cytokines, e.g. interferons or TNF, induced by CpG-injection, that are known to have anti-viral effects^20,21^, further support the therapeutic effect of the prime-boost vaccination. Since cytokines and in particular interferons are important for induction of CD8 T cells^23,24^, it is not possible to mechanistically dissect non-cytolytic cytokine-mediated from cytolytic control of HBV infection in this experimental setting of therapeutic vaccination in combination with CpG application. Induction of iMATEs through CpG-injection has also been shown to improve CD8 T cell immunity against high-level expression of a transgenic model antigen (ovalbumin) in the liver^25^, to increase anti-viral immunity during lymphocytic choriomeningitis virus infection^14^ and even increase anti-tumor immunity against liver cancer^26^. Such induction of increased T cell immunity is linked to hepatic recruitment of inflammatory monocytes that are functionally distinct from liver-resident macrophages^14,17^.

The combination of heterologous prime-boost vaccination to generate HBcAg-specific CD8 T cells in the first place is therefore nicely complemented by the subsequent local hepatic increase of HBcAg-specific CD8 T cells by CpG-mediated iMATE induction. Thus, our approach to apply first therapeutic vaccination followed by CpG injection combines two synergistic principles of immune therapy, i.e. first increasing numbers of HBcAg-specific CD8 T cells through potent cross-priming in the setting of a therapeutic vaccination and second amplifying these vaccine-induced HBcAg-specific CD8 T cells locally in the liver by CpG-mediated iMATE induction. Thus, iMATE-induced expansion of vaccine-induced HBV-specific CD8 T cells a may provide a liver-specific mean to increase the efficiency of therapeutic vaccination beyond currently available strategies^27^.

Although we cannot provide formal evidence for the role of GzmB expressed in HBcAg-specific CD8 T cells for elimination of HBV-infected hepatocytes, our finding that GzmB levels are increased in HBcAg-specific CD8 T cells after successful control of HBV-infected hepatocytes supports the notion that GzmB could be employed as biomarker for immune control of HBV-infection in future immune monitoring strategies during therapeutic vaccination against chronic hepatitis B. GzmB has been identified as essential downstream effector molecule for T cells to control viral infection^28^. GzmB expression has been shown to be higher in CX_3_CR1^+^ CD8 T cells with strong effector function^29,30^. The identification of GzmB^+^ HBV-specific CD8 T cells after therapeutic vaccination in combination with CpG injection therefore supports the notion that those T cells may have strong anti-HBV activity.

Taken together, our results demonstrate in two preclinical models of persistent HBV infection that the sequential combination of therapeutic vaccination followed by CpG-application was superior to vaccination alone in controlling persistent HBV infection by increasing numbers and functionality of HBcAg-specific CD8 T cells.

## Materials and Methods

### Ethical statement

Animal experiments were conducted in strict accordance with the German regulations of the Society for Laboratory Animal Science (GV-SOLAS) and the European Health Law of the Federation of Laboratory Animal Science Associations (FELASA). Experiments were approved by the District Government of Upper Bavaria (permission number: 55.2-1-54-2532-185-14). All efforts were made to minimize suffering.

### Experimental animals and AAV-HBV transduction

HBV transgenic mice (strain HBV1.3.32) carrying 1.3 overlength HBV (genotype D, serotype ayw) genome were created on C57BL/6 background (haplotype H-2^b/b^). Fourteen to sixteen weeks old female and male HBV transgenic mice were bred at the AVM Animal Facility, Helmholtz Center Munich. Wildtype C57BL/6 mice (haplotype H-2^b/b^) were purchased from Charles River Laboratories, Schulzfeld, Germany. Persistent HBV replication in wildtype mice was established by intravenous injection of 1 × 10^10^ genome equivalents (geq) of the AAV-HBV1.2 vector encoding 1.2-fold overlength HBV genome of genotype D, as reported prevsioulsy^18^. Mice were kept under pathogen-free (SPF) conditions at the Animal Facility, University Hospital Rechts der Isar, Technical University of Munich, or the Helmholtz Center Munich following institutional guidelines. Experiments were performed during the light phase of the day. Animals were bled one day before treatment and allocated into groups with comparable HBsAg and HBeAg levels.

### Heterologous protein prime MVA boost vaccination

Mice were immunized with a particulate protein prime followed by recombinant Modified Vaccinia Ankara virus (MVA) vector boost vaccination scheme described previously^10^. Briefly, mice were immunized intramuscularly (i.m.) into the quadriceps muscles of both hind limbs in isoflurane anesthesia. Protein immunization with 10 µg HBcAg expressed in *E. coli* (APP Latvijas Biomedicinas, Riga, Latvia) adjuvanted with 10 µg cyclic di-adenylate monophosphate (c-di-AMP) (InvivoGen, San Diego, CA) was given twice at 2-week interval. Two weeks after the second protein immunization, mice received 1 × 10^7^ particles of recombinant MVA expressing HBcAg intramuscularly (i.m.).

### CpG-application

20 µg CpG oligonucleotide (ODN 1668, InvivoGen, San Diego, CA) was applied intravenously. For control, mice were injected with 20 µg control non-CpG ODN (InvivoGen, San Diego, CA).

### Isolation of lymphocytes from spleen and liver and non-parenchymal liver cells

Preparation of single-cell suspensions of splenocytes was performed as described previously^31^. Liver-associated lymphocytes (LAL) were isolated by density gradient centrifugation as described^31^. Briefly, mouse liver was perfused with pre-warmed PBS (to flush blood from the hepatic vasculature) and forced through a 100 µm nylon cell strainer (BD Falcon, Franklin Lakes, NJ). After washing, cell pellets were suspended in 10 ml of prewarmed enzyme solution containing 1 mg/ml of collagenase type IV (Worthington, Lakewood, NJ) in RPMI 1640 medium supplemented with 10% fetal bovine serum (Gibco, Thermo Fischer Scientific, Darmstadt, Germany) and digested for 30 min at 37 °C. Cell pellets were then resuspended in 40% Percoll (GE Healthcare, Munich, Germany), layered on 80% Percoll solution and centrifuged at 1600 × g for 20 minutes without brakes for density separation. Non-parenchymal liver cells were obtained after liver perfusion, mechanical separation of liver tissue and gentle collagenase digestion for 10 minutes followed by percoll gradient centrifugation and washing steps as outlined in detail in^14^. Cell yield and viability were determined by trypan blue exclusion.

### Detection of HBcAg-specific CD8 T cells by multimers and intracellular staining and cell phenotyping by flow cytometry

MHC class I multimers conjugated with H-2K^b^-restricted HBcore peptide 93–100 (core_93–100_ MGLKFRQL) or ovalbumin-derived peptide (OVA_S8L_ SIINFEKL) were produced as published previously^32^ and kindly provided by D. Busch (Institute of Microbiology, TUM). Per sample, 0.4 µg streptamer was labelled with 0.4 µg Strep-Tactin-PE (IBA-lifesciences) in 30 µl FACS buffer and incubated for 30 min on ice. For intracellular cytokine staining, cells were stimulated overnight in the presence of 1 mg/ml Brefeldin A (Sigma-Aldrich, Taufkirchen, Germany) with synthetic HBcAg-derived peptide (HBc93), or Ovalbumin-derived peptide (SIINFEKL) added to a final concentration of 1 µg/ml. Cell surface staining was performed using anti-CD8 (clone 56.6–7; BD Biosciences, Heidelberg, Germany) and anti-CD4 (clone L3T4; BD Biosciences) T cell antibodies. Dead cells were excluded from analysis by Fixable Viability Dye eF780 (eBioscience, Frankfurt, Germany) staining. Intracellular cytokine staining was performed using Cytofix/Cytoperm Kit (BD Biosciences, Heidelberg, Germany) according to manufacturer’s instructions with anti-IFN-γ (clone XMG1.2; eBioscience) and cross-reactive anti-human granzyme B (clone: GRB04; Invitrogen, Carlsbad, CA) antibodies. Ki67 expression in T cells was detected using the antibody according to the instruction by the manufacturer (clone SolA15, eBioscience). Myeloid cell phenotyping was performed using CD11b (clone HK1.4) and MHC-II (clone M5/114.15.2) (BioLegend) and Ly6C (clone HK1.3), CD80 (clone 16-10A1), CD86 (clone GL1) antibodies (eBioscience). Data were acquired on a CytoflexS (Beckmann Coulter) flow cytometer. Analyses were performed using FlowJo software (Tree Star, Ashland, OR). Quantification of HBV-specific T cell numbers was performed with CountBright absolute counting beads (Invitrogen, Carlsbad, CA) following the manufacturer’s instruction.

### Serology

HBsAg and HBeAg titers were quantified in serum samples diluted 1:15 or 1:20 with phosphate buffered saline (PBS) on an Architect^TM^ platform using the quantitative HBsAg test (Ref.: 6C36-44) and the HBeAg reagent kit (Ref.: 6C32-27) with HBeAg quantitative calibrators (Ref.: 7P24-01) (all: Abbott Laboratories, Wiesbaden, Germany). Anti-HBs and anti-HBe antibodies were determined using anti-HBs (Ref.: 7C18-27) or anti-HBe (Ref.: 6C34-25) tests for an Architect^TM^ platform (Abbott Laboratories, Wiesbaden, Germany). ALT activity was measured in serum samples diluted 1:4 with PBS using Reflotron GPT/ALT tests (Roche Diagnostics, Mannheim, Germany).

### Immunohistochemistry

Liver tissue samples were fixed in 4% buffered formalin for 48 h and paraffin-embedded. Then, 2-μm-thin liver sections were prepared with a rotary microtome (HM355S, ThermoFisher Scientific, Waltham, USA). Immunohistochemistry was performed using a Bond Max system (Leica, Wetzlar, Germany, all reagents from Leica) with primary antibodies against Ki67 (clone SP6, Abcam ab16667) or rabbit anti-HBcAg primary antibody (Diagnostic Biosystems, Pleasanton, CA; 1:50 dilution) and horseradish peroxide coupled secondary antibody. Briefly, slides were deparaffinized using deparaffinization solution, pretreated with Epitope retrieval solution 1 (corresponding to citrate buffer pH6) for 20 minutes. Antibody binding was detected with a polymer refine detection kit without post primary reagent and visualized with DAB as a dark brown precipitate. Counterstaining was done with hematoxyline. Slides were scanned using a SCN 400 slide scanner (Leica Biosystems) and HBcAg-positive hepatocytes were determined based on localization, intensity and distribution of the signal in random 10 view fields (40x magnification). The mean numbers of HBcAg-positive hepatocytes were quantified per mm^2^.

### Statistical analyses

Statistical analyses were performed using GraphPad Prism version 5 (GraphPad Software Inc., San Diego, CA). Statistical differences were analyzed using Kruskal-Wallis test with Dunn’s multiple comparison correction, 2-way ANOVA with Tukey’s multiple comparison correction, Mann-Whitney test and unpaired or paired *t* test. *P*-values < 0.05 were considered significant.

## Supplementary information

supplementary figures and legends
