# Supplementary material for: Synergy of therapeutic heterologous prime-boost hepatitis B vaccination with CpG-application to improve immune control of persistent HBV infection
Source: Sci Rep. 2019 Jul 25;9:10808. doi: 10.1038/s41598-019-47149-w (PMC6658704; doi:10.1038/s41598-019-47149-w)
Supplement: Supplementary file 1 — supplementary figures and legends [file 41598_2019_47149_MOESM1_ESM.docx]

**Synergy of therapeutic heterologous prime-boost hepatitis B vaccination with CpG-application to improve immune control of persistent HBV infection**

Anna D. Kosinska^1,2,6^, Abdul Moeed^3^, Nina Kallin^3^, Julia Festag^1,2^, Jinpeng Su^1,2^, Katja Steiger^4^, Marie-Louise Michel^5^, Ulrike Protzer^1,2,6^*, and Percy A. Knolle^3,6^*

^1^Institute of Virology, Technical University of Munich; ^2^Helmholtz Centre Munich; ^3^Institute of Molecular Immunology and Experimental Oncology, Technical University of Munich; ^4^Institute of Pathology, Technical University of Munich; ^5^Institute Pasteur, Paris; ^6^German Center for Infection Research (DZIF), Partner site München

**Supplementary Figures**

**
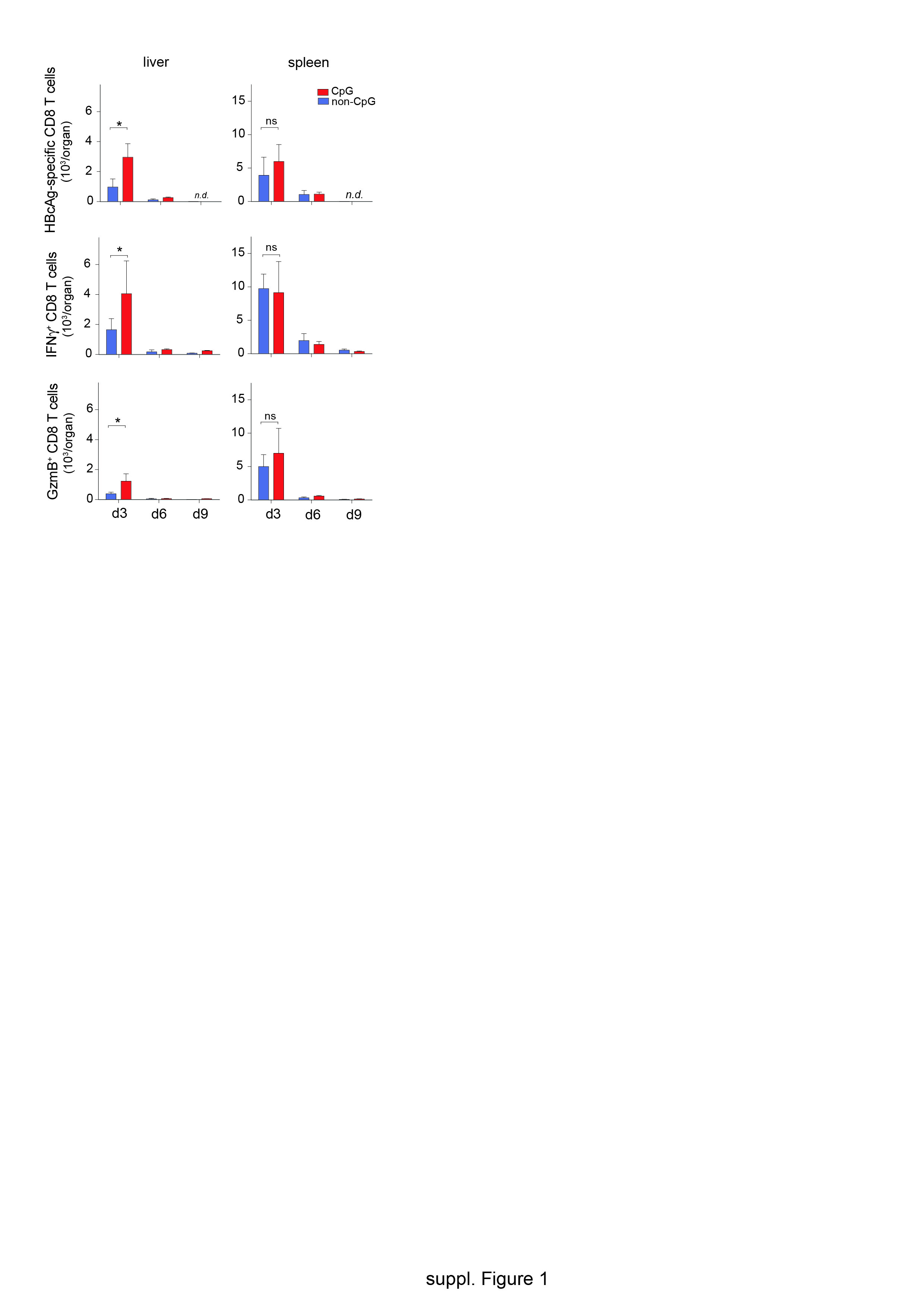
**

**Supplementary Figure 1: Optimal time point for CpG injection to enhance efficacy of prime-boost vaccination.** Wildtype C57BL/6 mice received heterologous prime-boost vaccination and at d3, d6 or d9 after the MVA boost mice received CpG or non-CpG control oligonucleotides. At d3 after the CpG injection, CD8 T cells were isolated from liver and spleen and numbers of HBcAg-specific CD8 T cells as well as IFNγ and GzmB expressing CD8 T cells after *ex vivo* stimulation with HBcore peptides were determined.

**
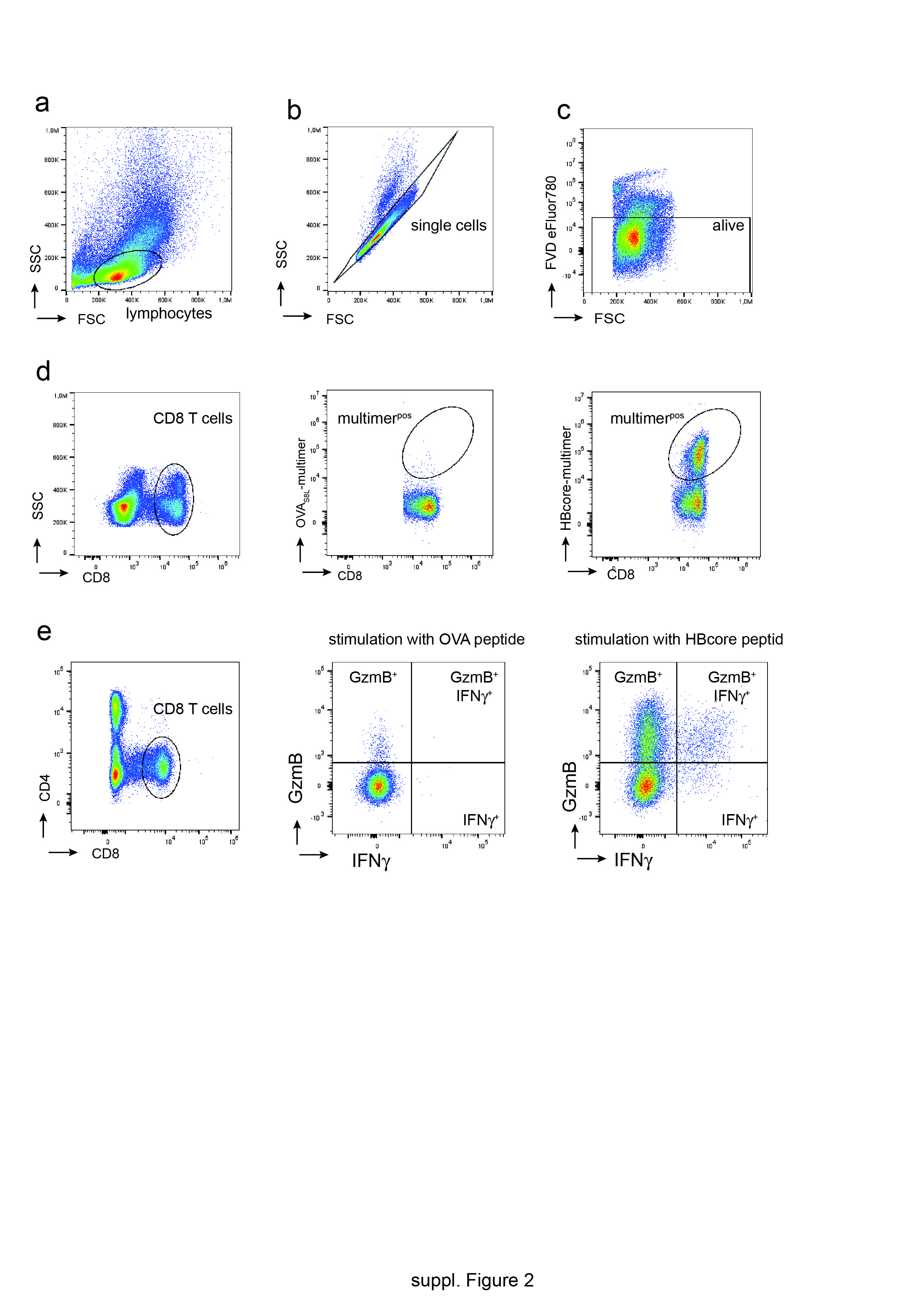
**

**Supplementary Figure 2: Gating strategy for detection of HBcAg-specific CD8 T cells**. (**a**) Lymphocyte populations were defined by flow cytometry using forward-scattered-light-height (FSC-H) and side-scattered-light-height (SSC-H) parameters. (**b**) Cell doublets were excluded from analysis by plotting forward-scattered-light height (FSC-H) against area (FSC-A) parameters and gating only on the diagonal population. (**c**) Dead cells were excluded from analysis by gating on fixable viability dye (FVD-eFluor780)-negative cells. (**d**) For multimer staining, CD8 T cells were gated within the viable lymphocyte population by plotting FSC-H against CD8-specific fluorescence signal. HBcAg-specific CD8 T-cells were then detected using fluorescently labelled HBcore-specific multimers. Positioning of the gate was determined using control cells stained with OVA_S8L_-specific multimers. (**e**) For intracellular cytokine staining, CD8 T cell populations were gated within the viable lymphocyte population by plotting CD4-specific fluorescence signal against CD8-specific fluorescence. To determine IFNγ and GzmB expression in response to *ex vivo* stimulation with HBcore peptides, HBcAg-reactive CD8 T cells were detected by intracellular cytokine staining; to exclude non-specific staining gates for analysis were set using CD8 T cells stimulated with ovalbumin-derived peptides (OVA_S8L_; SIINFEKL).


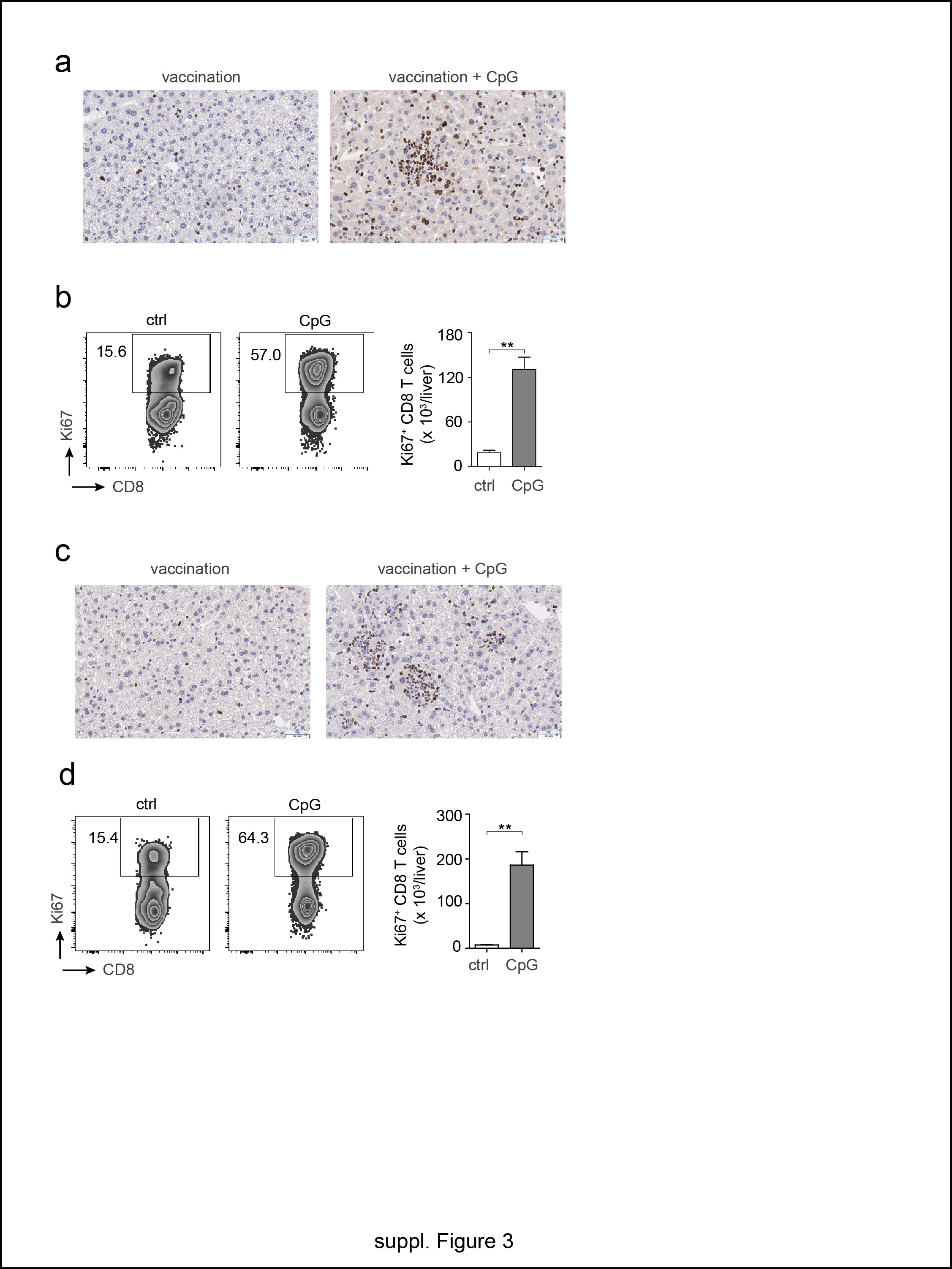


**Supplementary Figure 3**: **Expression of Ki67 in hepatic immune cells after CpG injection**. (**a**) Immunohistochemistry for Ki67 expression in liver slices of vaccinated HBV-transgenic mice at d3 after CpG injection. (**b**) Ki67 expression detected by flow cytometry in hepatic CD8 T cells from HBV-transgenic mice, shown in a representative dot plot (left panels) and as quantification of numbers of positive CD8 T cells (right panels). (**c**) Immunohistochemistry for Ki67 expression in liver slices of vaccinated AAV-HBV transduced mice at d3 after CpG injection. (**d**) Ki67 expression detected by flow cytometry in hepatic CD8 T cells from AAV-HBV transduced mice, shown in a representative dot plot (left panels) and as quantification of numbers of positive CD8 T cells (right panels). Bars in (b,d) indicate mean values of n ≥ 3 mice per group + SEM. Statistical analyses were performed using Student´s t test. Asterisks mark statistically significant differences: ** *p* < 0.005.


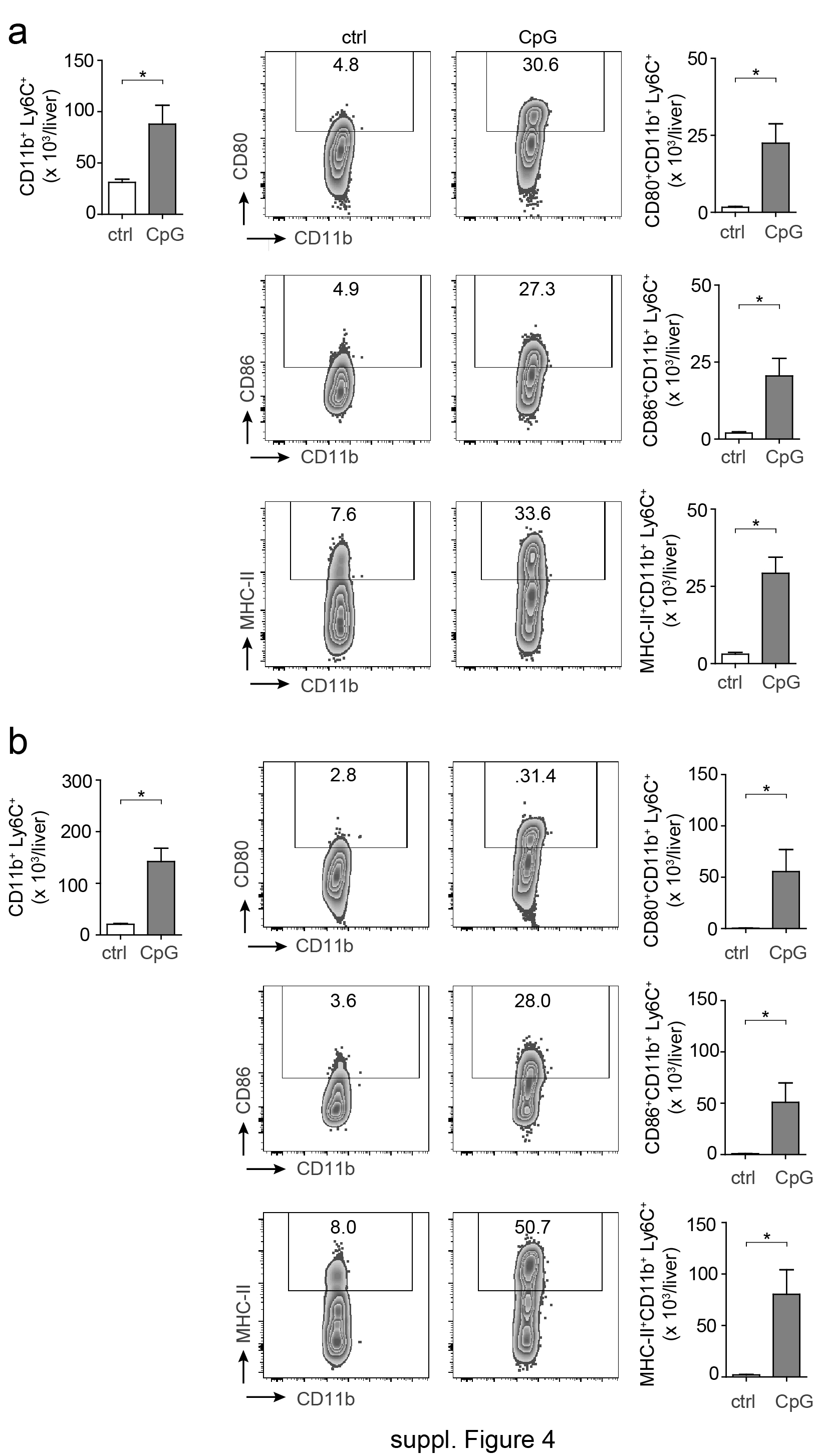


**Supplementary Figure 4**: **Phenotypic characterization of liver CD11b^+^ cells after CpG injection**. (**a**) Non-parenchymal cells were isolated from ctrl or CpG-injected HBV transgenic mice (d3 after CpG injection) and subjected to flow cytometric analysis. Shown are: increase in total numbers of CD11b^+^Ly6C^+^ inflammatory monocytes (left panel); augmented surface expression levels of CD80, CD86 and MHC-II in representative dot plots (middle panels) and quantification of the numbers of positive cells (right panels). (**b**) Analysis identical to (a) but from AAV-HBV transduced mice. Bars indicate mean values of n ≥ 3 mice per group + SEM. Asterisks mark statistically significant differences: * *p* < 0.05.


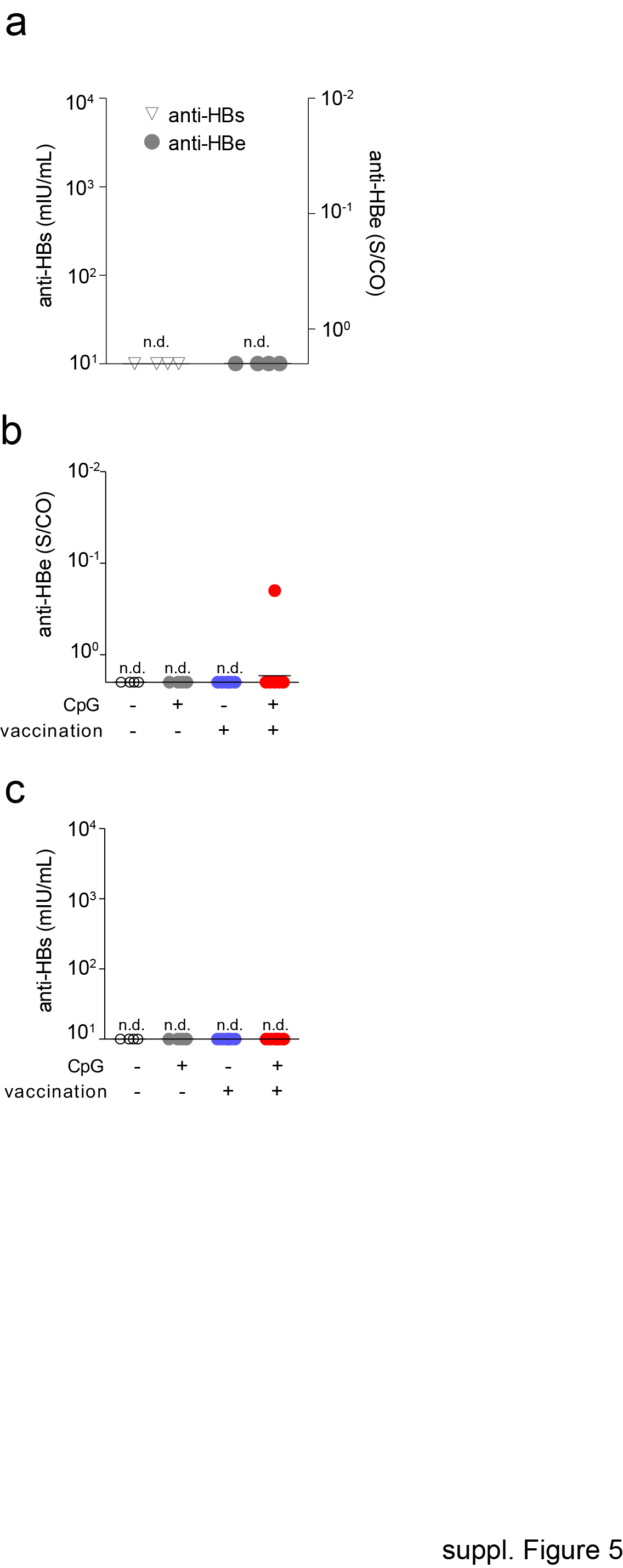


**Supplementary Figure 5**: Lack of anti-HBs or anti-HBe seroconversion. (**a**) Results for anti-HBs and anti-HBe antibody detection in serum of AAV-HBV transduced mice before start of vaccination. (**b,c**) Evaluation of anti-HBe (**b**) and anti-HBs antibodies (**c**) in serum from AAV-HBV transduced mice at d63. *n.d*. – not detectable.
